# A call to readjust the Israeli school feeding program

**DOI:** 10.1186/s13584-023-00568-7

**Published:** 2023-05-10

**Authors:** Dorit Nitzan

**Affiliations:** School of Public Health, Director of Masters Program in Emergency Medicine- Preparedness and Response and Chair, Food Systems, One Health and Resilience, Ben Gurion University of the Negev, Beer Sheva, Israel

**Keywords:** School feeding program, Food security, One Health, Emergencies, Child health

## Abstract

The COVID-19 pandemic challenged the food and nutrition security status of thousands of children in Israel. This commentary argues that policymakers should urgently readjust the Israeli school feeding program based on experts’ advice. Children should have the right to select food items, grow the items, prepare the meals, and clean and care for the waste together. They should eat as a community in suitable school dining rooms. Access to the school feeding program should also be ensured during emergencies, school closures, isolation and quarantine, treatment, and rehabilitation of children. The food provided through the program should be integrated into the food baskets of their families, aimed at improving their households’ food and nutrition security. It is important to activate a universal school feeding program that does not differentiate, separate, and stigmatize children, their households, their communities, and their schools. The United States National School Lunch Program is briefly reviewed, highlighting the importance of the program's routine monitoring, evaluation, and improvement. Engaging the children in planning the meals and in the production, preparedness, provision, and waste management processes are key to improving their involvement, health literacy and promotion, and their families’ resilience. Implementing a holistic Food System Approach, including school gardening and “Farm to School,” is suggested. It is recommended to urgently formulate a modern, universal, and comprehensive Israeli Food and Nutrition Security Plan, with a dedicated chapter for the upgraded School Feeding Programe with a section on its implementation in emergency preparedness, response, and Resilience. It should be anchored in the Food Systems framework and the One Health Approach.

## Learning from the COVID-19 pandemic—food security and resilience

In their important paper on child food insecurity in the wake of the COVID-19 pandemic—Azarieva, Berry, and Troen have outlined the most urgent three actions for the Israeli school feeding program (SFP). First, explicitly state the goal to address child insecurity in the SFP law; request that the government assume responsibility for the routine assessment and data collection on food insecurity among Israeli children; and integrate a monitoring and evaluation program. They also emphasize the need to provide a “universal” SFP to improve the health of all Israeli children across all socioeconomic backgrounds [1].

The COVID-19 pandemic has revealed gaps and weaknesses in our social, health, and education systems, including governance, finance, resources, communication, community engagement, and service delivery. The current commentary supports and adds a few more dimensions to the authors’ call and highlights the importance of SFPs and their role in ensuring food and nutrition security in the prevention of preparedness for and response to health emergencies. Many countries, including Israel, had functioning targeted SFPs before the pandemic. With school closures, these programs were mostly put on hold, risking the food and nutrition security of children who depend on these meals. Many of them were challenged since they missed school days due to their isolation and/or quarantine, and their families’ livelihoods were adversely affected. When the SFPs were needed the most—they were not available and accessible to those in need.

## SFP in Israel

The Israeli SFP, known as the “Hot Lunch Program,” provides hot meals to some children in schools throughout the country. There are three SFP programs: 1. Nitzanim—accessible to kindergarten children and first and second graders enrolled in “after-school programs.” It is provided only by the local authorities with the lowest socioeconomic status. This program covers about 240,000 children; 2. Daily Meal for Pupils—following the respective law of 2005, children in kindergartens and elementary schools who are part of the “long learning days” are eligible for a hot meal per day. The local authorities are responsible. In 2001 306,000 children were eligible, but only 190,000 were enrolled; and 3. MILAT Program—for children enrolled in extended school days who are new immigrants or those who reside in the periphery, their school or kindergartens do not implement long school days. It is thus a selective program. The Ministry of Education mainly funds the SFP. The participation of the local authorities and the parents in each of the programs is differential and is set by the socio-economical cluster [2].

Even before the pandemic, the SFP had been criticized for not aiming at food insecurity, particularly among low-income families. It is important to note that many children need access to the SFP. For example, only a small number of high school children are covered by MILAT. Also, the 2005 SFP law only covers SFP in schools enrolled in the “Long School Day” program. Also, children from disadvantaged families who reside in higher socio-economic clusters are not included in the SFP. These gaps and the lack of a universal childhood feeding program resulted in the provision of food by some civil society organizations (CSOs) and support by some local authorities. It also resulted in the submission of new proposed draft laws, among them the inclusion of students in 7–11 grades, the inclusion of all schools that work for more than 37 h per week, the provision of meals to children during their vacation and periods where schools are closed, provision of meals to high school children and making the SFP universal [2].

It is also important to note other aspects of the Israeli SFP: the meals are prepared, packed, and delivered to the schools included in the SFP by contracted catering companies. Some concerns have been raised regarding how the meals are handled and served to the children, as well as the wasted food, boxes, and utensils. There have been reports of meals that do not fit nutritional needs and/or fit into the culinary culture of some children. Lunch is served in most schools in the classrooms, often missing appropriate ventilation, hygiene, and sanitary services. Moreover, the children are regarded as passive consumers as they are usually not engaged in the decision on the menu construction, do not have the option to select food items, and are not involved in the food production and preparedness processes.

Nevertheless, considering all the challenges mentioned above, the Israeli SFP could serve as an important feeding source for children during routine and emergencies. For this, it is recommended to urgently formulate a modern, universal, and comprehensive Israeli Food and Nutrition Security Plan, with a dedicated chapter for the upgraded SFP, outlining the actions to be taken during emergency preparedness, response, and resilience. It should be anchored in the Food Systems framework and the One Health Approach.

## Children-centered universal meals as a leverage for health and human capital

‘Food security’ exists when all people, at all times, have physical, social, and economic access to sufficient, safe, and nutritious food to meet their dietary needs and food preferences for an active and healthy life. It comprises four pillars: availability, accessibility, utilization, and stability. It does not include food production, food consumption, and nutrition status [3]. Therefore, the term Food and Nutrition Security (FNS, e.g., “when all people at all times have physical, social and economic access to food which is consumed in sufficient quantity and quality to meet their dietary needs and food preferences and is supported an environment of adequate sanitation, health services, and care, allowing for a healthy and active life”) should be used. All the FNS dimensions are integrated into sustainable Food Systems. A sustainable food system delivers food security and nutrition for all so that the economic, social, and environmental bases to generate food security and nutrition for future generations are not compromised [4]. In Israel, Food and Nutrition insecurity is linked to poverty and was documented and brought to policymakers in 2003 [5].

FNS are prerequisites to improve the nutritional status, immunity, health, well-being, and physical and cognitive functions of children now and in their future [6, 7]. The provision of universal, children-centered SFPs would remove the risks for discrimination and stigmatization of underprivileged children due to their separation from the rest of the groups while tailoring to the children’s food preferences that contribute to healthy nutritional intake. Involving the children in the production of the food items (e.g., growing vegetables and fruit in schools), management of procurements and decision making, as well as preparing the food, serving it, cleaning and managing the waste would make the children agents of health for the whole community. Eating together in school dining rooms would promote community life, inclusion, respect, and a healthier environment. Such food system-related activities combined with eating in health-promoting settings will contribute to social togetherness and cohesion, learning and adapting to different culinary cultures while promoting good health and nutrition. In this way, the SFPs achieve a much broader objective. Universal and children-centered SFPs should continue beyond schools and become an integral part of the household’s food and nutrition security schemes.

## The United States national school lunch program

The United States of America (USA) National School Lunch Program began in 1946 and was joined by the School Breakfast Program in 1966. Both constitute the School Meal Program administered by the United States Department of Agriculture. They are based on compulsory nutrition standards for school food (including snacks) that must reflect the most recent Dietary Guidelines for Americans. Nutrition education is an integral part of the programs. The programs went through changes. The “Farm to School” approach has brought fresh produce into schools while linking the pupils with local producers. Many children are now enrolled in breakfast, schools, and after-school feeding programs; the SFP supports the local growers; professionals with the children tailor the meals; and during the COVID-19 pandemic, the meals arrived to the children in many locations [8].

During the pandemic, some schools continued operating remotely, but with support from the federal budget, the SFP was ready to reach the children. Some meals and/or food items could be collected or delivered to the children’s homes. In addition, a new Pandemic Electronic Benefits Transfer (P-EBT) Program provided the families of school children with debit-type cards that could be used to purchase school meal items in stores.

## SFPs monitoring and evaluation

Monitoring and evaluation (M&E) of the SFPs is important to reveal their effectiveness, guide their improvement and ensure a positive impact on the health and well-being, health and nutrition status. Many countries carry out M&E routinely, including Finland, France, Japan, and the United States.

As mentioned, it is important to institute routine M&E in Israel [1]. The comprehensive tool should assess the children’s food and nutrition security, food safety, processes, systems, structures, environmental impact, community, and children’s engagement. Various indicators such as satisfaction, enrollment, attendance, retention, learning outcome, and nutritional status should be included. The reports with the findings and the ways forward should be transparent and accessible. Furthermore, they should be incorporated into routine area- and population-based food and nutrition security and One Health surveys.

## Wisdom from hindsight: food and nutrition security from the pandemic to climate change and resilience

Among the 183 governments that responded to the Global Child and Nutrition Survey in 2001, 90% of the reported that their SFP aims to meet the children's nutritional and/or health needs. About a third of them mentioned their goal to prevent/ mitigate obesity (more common among high-income countries) [9].

In many countries, SFPs faced the challenge of making meals accessible to children. About 78 out of 134 national governments that responded to the Global Child and Nutrition Survey indicated that “most” schools were either closed, operating remotely, or used some hybrid status for at least one month in the school year that began in 2020. 38% of the governments indicated that schools were not open for in-person learning for at least six months.

The bi-directional impact of environment and climate change on food systems is associated with a heavy impact on global Food and Nutrition Security. The food items selected for SFP, transportation, packaging, and waste heavily affect the environment. Thus, it is important to equip our current and future generations with the tools to prevent, prepare for, and adapt to environmental challenges. The SFPs serve as the most natural platforms for such educational acts and equip the children with practical and useful tools. It is important to promote the Mediterranean Diet also through the SFPs. By bringing the full Food System onto the stage, the children could promote and participate in home and school gardening, learn to think out of the box and look for solutions, innovate, and practice indoor smart agriculture and beyond [10]. This is a good opportunity to integrate the TNUFA action plan (Adequate Nutrition for the Promotion of Human Capital), which was engineered already in the early 2000s (personal documents), into such comprehensive policies.

## Conclusions

This commentary recommends formulating a modern, universal, and comprehensive Israeli Food and Nutrition Security Plan. It should include a chapter on SFP that is children-, household- and community-centered during peaceful and emergency times. It should be based on the Mediterranean Diet and anchored in the Food Systems framework and the One Health Approach. In this way, the delicate balance between the health of people, animals, plants, and the environment will be considered and managed from the outset. The SFP should be monitored and studied to ensure its positive impact on the nutritional status of children and their families.

## Data Availability

Not applicable. No data was used for this commentary, and all references were provided.

## Abbreviations

CSO: Civil Society Organization
M&E: Monitoring and evaluation
SFP: School feeding program
USA: United States of America
